# Short-term reproductive outcomes analysis and prediction of the modified uterine stent treatment for mild to moderate intrauterine adhesions: experience at a single institution

**DOI:** 10.1186/s12905-024-03098-9

**Published:** 2024-04-23

**Authors:** Chaoxia Cao, Yinan Chen, Jinjin Li, Qianjie Xu, Xiaoli Liu, Ruikun Zhao, Quanjia Jiang, Qin Zhou

**Affiliations:** 1 Department of Obstetrics and Gynecology, Women and Children’s Hospital of Chongqing Medical University, 401147, Yubei District, Chongqing, China; 2 Department of Obstetrics and Gynecology, Chongqing Health Center for Women and Children, 401147, Yubei District, Chongqing, China; 3 Chongqing Research Center for Prevention and Control of Maternal and Child Diseases and Public Health, 401147, Yubei District, Chongqing, China; 4 Department of Mathematics, School of Mathematics and Physics, University College London, Gower St, London, WC1E 6AE UK; 5 Department of Obstetrics and Gynecology, the First Affiliated Hospital of Chongqing Medical University, Yuzhong District, Chongqing, 400016 China; 6 Department of Health Statistics, School of Public Health, Chongqing Medical University, Yuzhong District, Chongqing, 400016 China; 7 Department of Obstetrics and Gynecology, Chongqing Shapingba Maternity and Child Healthcare Hospital, Shapingba District, Chongqing, 401331 China

**Keywords:** Intrauterine adhesions, Hysteroscopy, Modified uterine stent, reproductive outcomes, endometrial thickness

## Abstract

**Background:**

To evaluate the efficacy of modified uterine stent in the treatment of mild-to-moderate intrauterine adhesions and explore the relative indicators affecting prognosis prediction.

**Methods:**

A total of 115 patients with mild-to-moderate intrauterine adhesions received a modified uterine stent placement after hysteroscopy adhesiolysis. The second-look hysteroscopy operated after 3 months surgery, and the third-look hysteroscopy operated after 6 months surgery if necessary. The stent was removed when the cavity shape was repaired, then the reproductive outcomes were followed up one year.

**Results:**

Menstrual blood volume, endometrial thickness and volume had increased significantly after 3 months surgery. The rates of cavity repaired were 86.96% (100/115) after 3 months surgery and 100% (115/115) after 6 months surgery cumulatively. Endometrial thickness after 3-months surgery was positively associated with uterine cavity shape repaired (*P*<0.01). The receive operating characteristic (ROC) curve showed the rate of uterine cavity shape repaired predicted by the model was 0.92, based on the endometrial thickness after 3-months surgery. The rate of pregnancy was 86.09% (99/115) in one year, while the rate of miscarriage accounted for 26.26% (26/99). The median time interval between stent removal and subsequent conception was 3 months. It showed adhesion recurrence was the risk factor for subsequent pregnancy (*P*<0.01).

**Conclusions:**

A modified uterine stent placement under hysteroscopy was an effective approach for mild-to-moderate intrauterine adhesions, which is easy to operate and worthy for clinical promotion. Endometrial thickness measured by ultrasonography probably has predictive value in adhesion recurrence and subsequent pregnancy.

**Trial Registration:**

ChiCTR2100051524.

Date of registration (retrospectively registered): 26/09/2021.

## Introduction

Intrauterine adhesions (IUAs), known as Asherman syndrome, have been first reported in 1894 [1]. It is the scarring disease in essence, characterized by uterine cavity narrowed and thin endometrium [2–4]. IUAs may be associated with abnormal menstruation, recurrent pregnancy loss, secondary infertility, and pregnancy complications [5–8]. In recent years, the incidence of IUAs has increased worldwide as a result of the high rate of induced abortion and the improvement of diagnostic techniques, such as three-dimensional ultrasonography and office hysteroscopy [9, 10].

Hysteroscopic adhesiolysis is the optimum route for treatment of IUAs [9, 11–13]. However, the rate of recurrence is 30% to 66% [12–14]. It is a knotty problem to prevent adhesion recurrence after surgery. To date, many approaches have become available, including cross-linked hyaluronic acid gel, balloon catheter and intrauterine device (IUD) [12, 15, 16]. There is still no consensus regarding the optimal postoperative treatment of IUAs [17]. In our hospital, IUAs has been treated with hysteroscopic procedures for more than 20 years. This retrospective study aimed to report our experience with a modified uterine stent placement under hysteroscopy treatment of mild-to-moderate IUAs and describe the short-term outcomes.

## Materials and methods

### Ethical approval

Patients who were diagnosed with mild-to-moderate IUAs treated with the modified uterine stent placement after hysteroscopy adhesiolysis in outpatient from October 2020 to March 2021 were retrospectively studied. The study protocol was approved by the Ethics Committee of the first affiliated Hospital of Chongqing Medical University (approval number 2020-572). Informed consent was obtained from all participants.

### Study design

Adult women with hypomenorrhea, infertility, or recurrent spontaneous abortion were confirmed by office hysteroscopy. The eligibility criteria were women with strong pregnancy desire and diagnosed as mild (1-4 points) or moderate (5-8 points) IUAs with the American Fertility Society (AFS) classification of intrauterine adhesions [18]. The exclusion criteria were as follows: infertility caused by tubal, endocrine or male factors; congenital uterine malformation; IUAs caused by endometrial tuberculosis or uterine artery embolism; and diseases with submucous myoma, adenomyosis, or endometrial polyp.

**Fig. 1:**
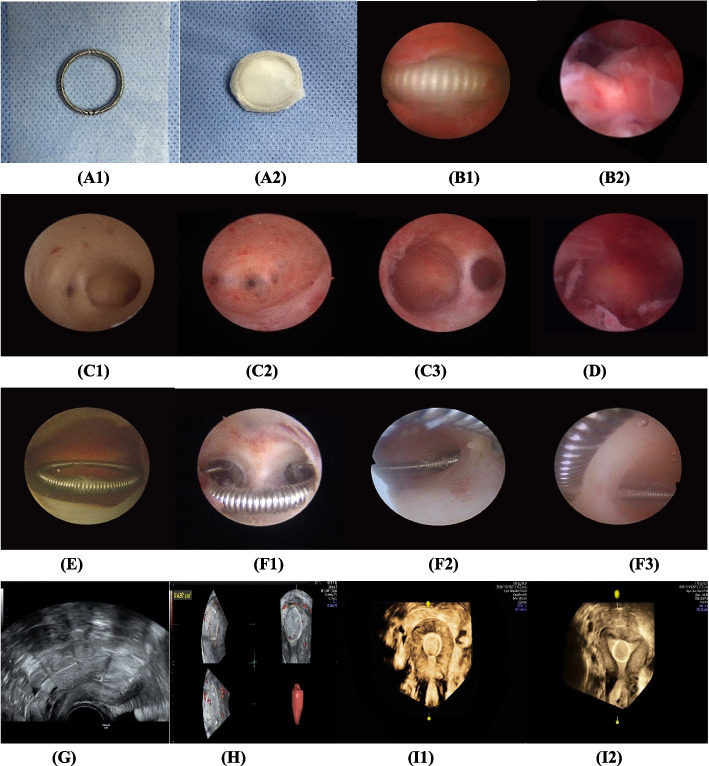
The process description of treatment and ultrasonography: A1-2 A round-shaped stainless-steel IUD packed by an anti-adhesive membrane of Chitosan; B1-2 The modified uterine stent was in uterine cavity; C1-3 The types of IUAs; D The uterine cavity after adhesiolysis immediately; E The uterine cavity and the stent (Chitosan membrane had been degraded) at the second-look hysteroscopy; F1-3 Adhesion recurrence appeared as device incarceration at the second-look hysteroscopy; G The endometrial thickness measured by 2D- TVUS; H The endometrial volume measured by 3D- TVUS; I1 It prompted the stent incarcerated, I2 It prompted the cavity repaired well without the stent incarcerated. 2D-TVUS: two-dimensional transvaginal ultrasound; 3D-TVUS: three-dimensional transvaginal ultrasound

Two senior surgeons using 5.5 mm diameter hysteroscope (Stryker, American) performed all operations. All cases received adhesiolysis by scope or minisize scissor to restore the anatomy of the uterine cavity, then a modified uterine stent was placed immediately into uterine cavity to maintain the cavity shape. The stent was made from a round stainless-steel containing copper (OCu200-21, Wuxi Tianyi Medical devices Co. LTD, China) packed by an anti-adhesive membrane of Chitosan (Guangzhou Hong Jian Bio-Medical Products Co. LTD, China) (Fig. 1). The second-look hysteroscopy operated after 3 months surgery. When the stent was embedded in adhesive tissue, we called it as stent incarceration or adhesion recurrence, it considered the cavity shape was not repaired, the new stent was replaced for another three months, then the third-look hysteroscopy operated; when there was no stent incarceration, it considered the uterine cavity shape was repaired, and the stent was removed.

Endometrial thickness and endometrial volume were measured by transvaginal ultrasound (TVUS) (GE VOLUSON E8, USA) during the mid-luteal phase of patients’ menstrual cycle. Menstrual blood volume and pregnancy outcomes were collected. The termination time of follow-up was at July 1, 2022. Only data providing from women with the persistent pregnancy desire and completed the whole procedure were finally included in the analysis.

### Statistical analysis

Statistical analyses were performed with SAS 9.4. Continous data were presented as mean±SD for normal distribution and medians (interquartile ranges) for skewed distribution. Continuous data were compared by Student’s t-test, analysis of variance, non-parametric test, or the Kruskal-Wallis test, as appropriate. Categorical data were expressed as frequencies and compared by chi-square test. Logistic regression analysis was used to explore the specific relationship between variables. The receiver operating characteristic (ROC) curve analysis and Youden index were used to predict uterine cavity restored and subsequent pregnancy. All tests were two-tailed, and *P*<.05 was considered to indicate statistical significance.

## Results

**Fig. 2:**
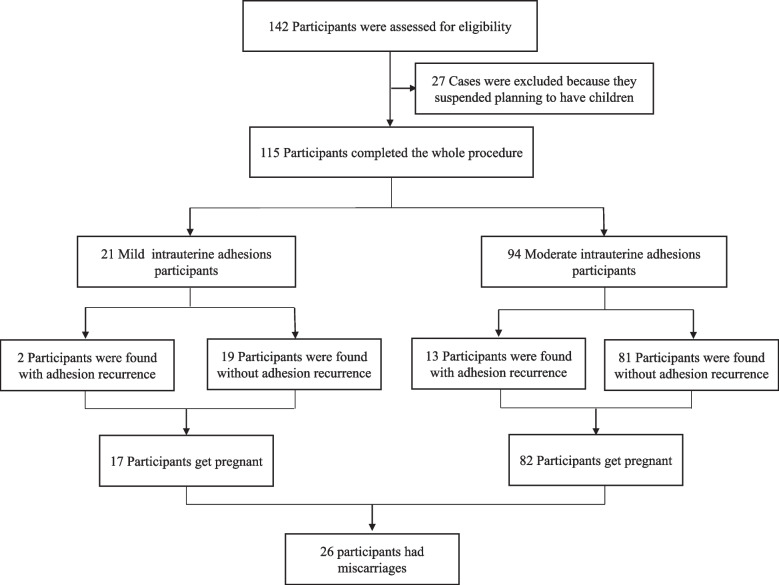
Distribution of outcome

From October 2020 to March 2021, 142 patients underwent the whole procedures for eligibility without perforation, infection and other complications. 27 patients were excluded, because they suspended planning to have children. 115 patients (age 31.03±4.07 years, gravidity 2.77±1.48 times, Parity 0.43±0.58 times) were included in the analysis. Among them, the mild IUAs accounted for 18.26% (21/115), and the moderate IUAs accounted for 81.74% (94/115), respectively (Fig. 2).

### Clinical Characteristics before and after 3 months surgery

70 (60.87%) cases got the menstrual blood volume increased. Endometrial thickness and endometrial volume (Fig. 1) had significantly increased after 3-months than those before surgery (*P*<0.0001) (Table 1).

**Table 1:** The endometrial thickness and volume before surgery were compared with those after 3 months surgery

	**Before surgery** **(mean ±SD)**	**After 3-months surgery** **(mean ±SD)**	**D-value** **(mean ±SD)**	**P**
Endometrial thickness (mm)	6.57±2.07	7.87±1.74	1.30±2.15	<.0001^a^
Endometrial volume (cm^3^)	2.02±1.06	2.75±1.08	0.73±0.78	<.0001^a^

*SD* standard deviation, *D-value* Difference value

^a^Paired sample sign rank test

### Hysteroscopy and Ultrasonography Characteristics at the second or third look

The shape of uterine cavity and the stent position had been evaluated by ultrasonography and hysteroscopy (Fig. 1). The rates of cavity repaired were 86.96% (100/115) at the second look hysteroscopy and 100% (15/15) at the third look hysteroscopy cumulatively. 15 (13.04%) cases with adhesion recurrence had stent incarcerated in the newly formed scar at the second-look hysteroscopy. However, their uterine cavities were all maintained at the third-look hysteroscopy.

**Fig. 3:**
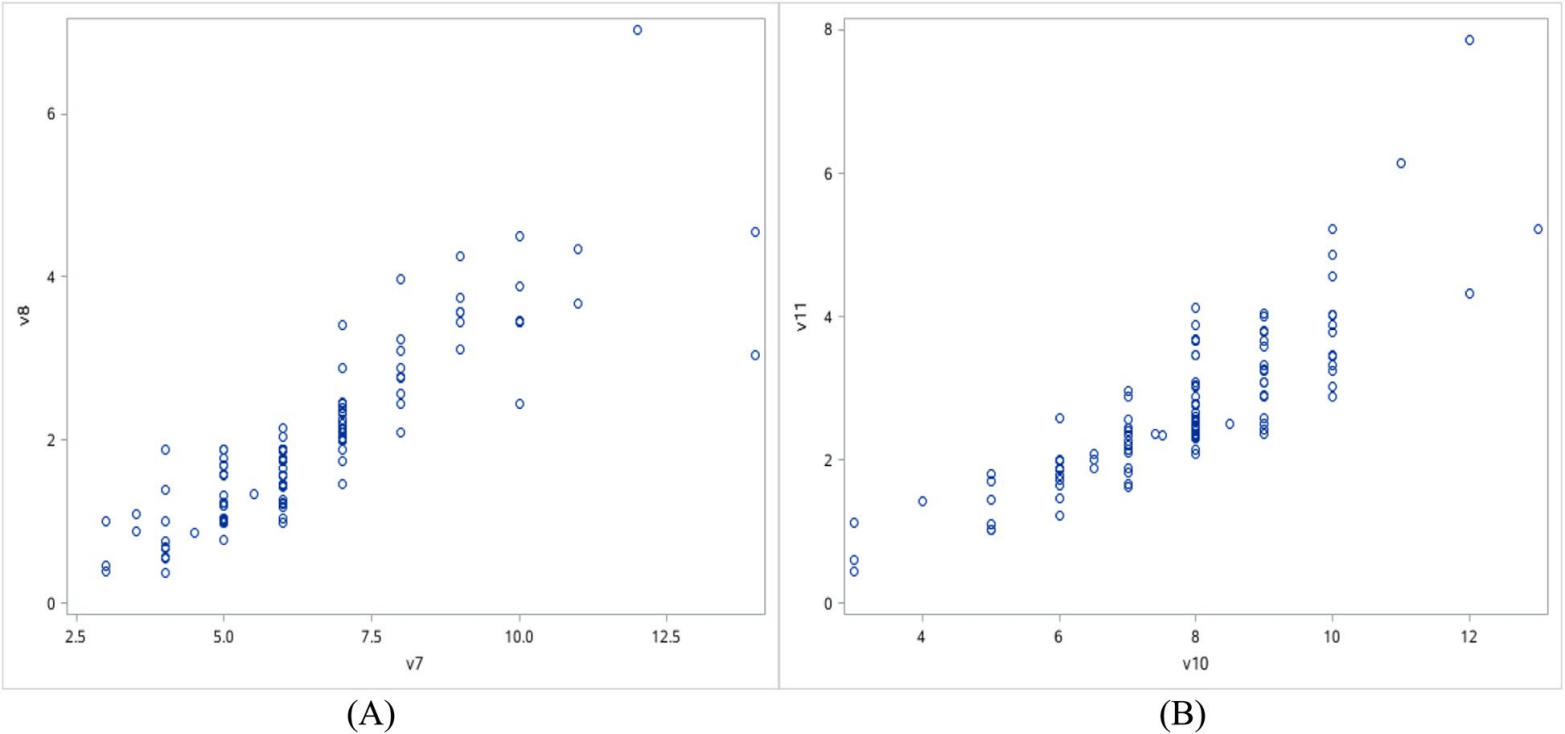
Scatter plots of endometrial thickness and volume: A Scatter plots of endometrial thickness and volume before surgery. B Scatter plots of endometrial thickness and volume after 3-month surgery

**Fig. 4:**
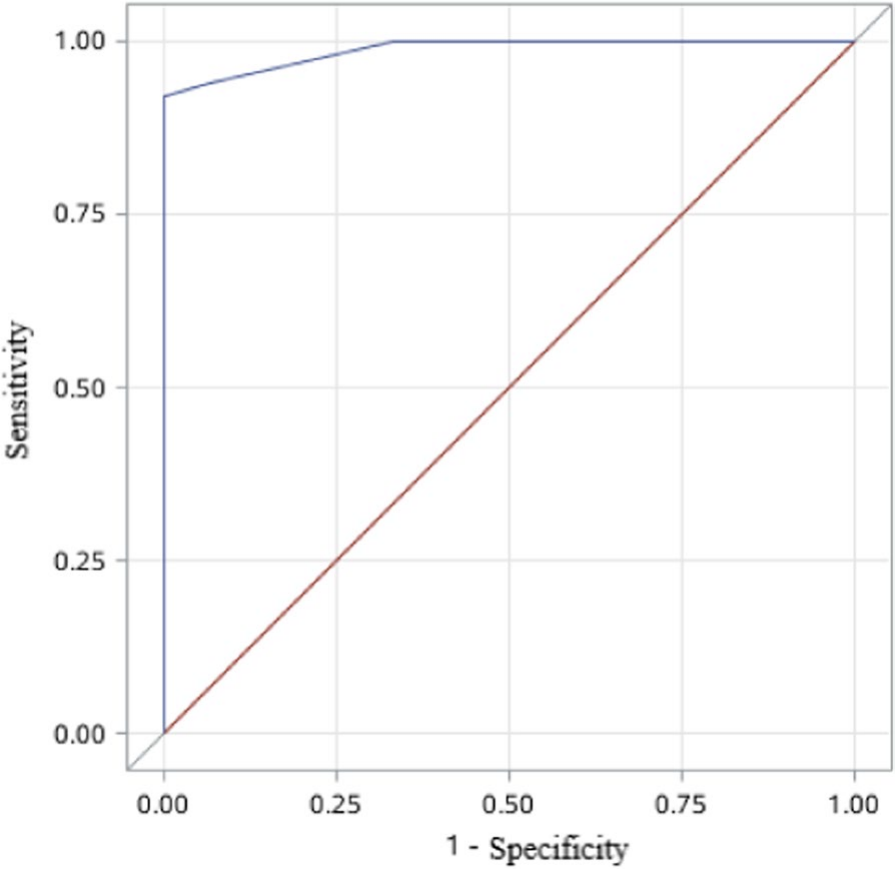
The ROC curve of the endometrial thickness predication model for uterine cavity shape repaired at the second look hysteroscopy: It predicted by the model is 0.92 (area under the curve =0.9873; sensitivity = 92.0%; specificity = 100%) based on endometrial thickness after 3-month surgery when the endometrial thickness was 7 mm

The endometrial thickness and volume measured before and after 3-months surgery were significantly greater in patients with uterine cavity shape repaired than those in patients without repaired at the second-look hysteroscopy (*P*<0.01) (Table 2). Stepwise logistic regression analysis showed that endometrial thickness not endometrial volume measured after 3-months surgery was positively associated with uterine cavity shape repaired (*P*<0.01) (Table 3). Despite the scatter plots showed that endometrial thickness and volume these were significant correlation (Fig. 3). The ROC curve showed that the model of endometrial thickness measured after 3-months surgery predicted the rate of uterine cavity shape repaired was 0.92 when it was 7 mm (Fig. 4).

**Table 2:** Comparison general conditions in patients with and without uterine cavity repaired at the second look hysteroscopy

**Project**	**Cavity repaired** **(*n*=100)**	**Cavity non-repaired (*n*=15)**	**Rate of cavity repaired (%)**	**P**
Adhesion degree before surgery				
Mild	19	2	90.48	0.8639^a^
Moderate	81	13	86.17	
Menstrual blood volume before surgery				
Hypomenorrhea	66	13	83.54	0.1899^a^
Normal	34	2	94.44	
Endometrial thickness before surgery (mm)	7.00(5.00,8.00)	5.00(4.00,6.00)		0.0016^b^
Endometrial volume before surgery (cm^3^)	1.93(1.42,2.51)	1.23(0.76,1.57)		0.0005^b^
Endometrial thickness after 3-months surgery (mm)	8.00(7.75,9.00)	5.00(4.00,6.00)		<.0001^b^
Endometrial volume after 3-months surgery (cm^3^)	2.64(2.35,3.46)	1.43(1.02,1.78)		<.0001^b^

^a^Continuous correction chi-square test

^b^Nonparametric test

**Table 3:** Analysis of affecting factors for uterine cavity shape repaired

	**Univariate logistic regression analysis**			**Stepwise logistic regression analysis**		
**Parameter**	***P*-value**	**OR**	**95%CI**	***P*-value**	**OR**	**95%CI**
Endometrial thickness before surgery	0.0050**	1.806	1.195-2.730			
Endometrial volume before surgery	0.0021**	4.673	1.750-12.48			
Endometrial thickness after 3-months surgery	0.0084**	59.835	2.854-1254	0.0084**	59.835	2.854-1254
Endometrial volume after 3-months surgery	0.0004***	723.761	18.67-28059			
Adhesion degree before surgery	0.5986	0.656	0.136-3.153			
Menstrual blood volume before surgery	0.1254	3.347	0.714-15.69			

^**^P<0.01

^***^P<0.001

### Short-term pregnancy outcomes

**Fig. 5:**
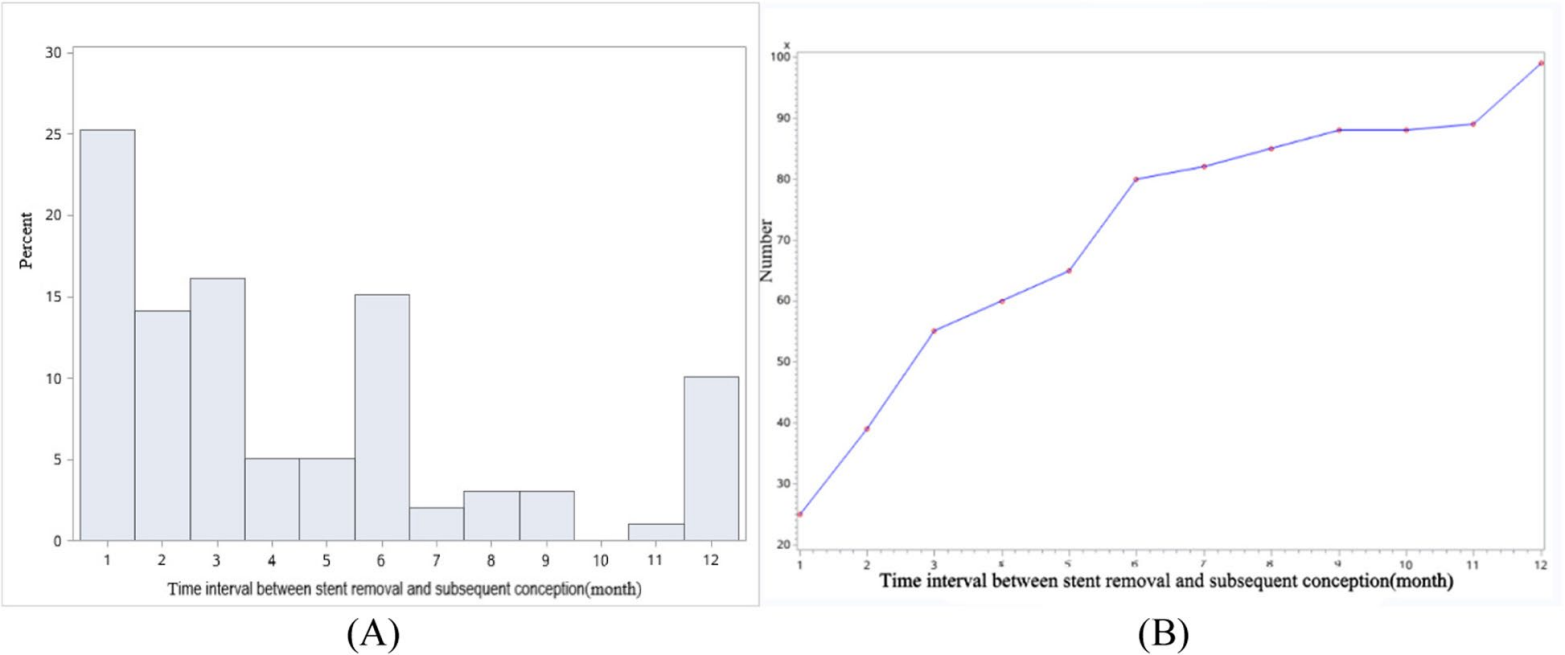
The time interval between stent removal and subsequent conception distribution within one year:** A** The number and time interval distribution of conception; **B** Cumulative conception distribution with time interval

The median time interval between stent removal and subsequent conception was 3 months (ranged from 1 to 12 months). 99(86.09%) patients had pregnancy spontaneously, no patient got more than one conception during one year of follow-up (Fig. 5), while the rate of miscarriage accounted for 26.26% (26/99).

**Fig. 6:**
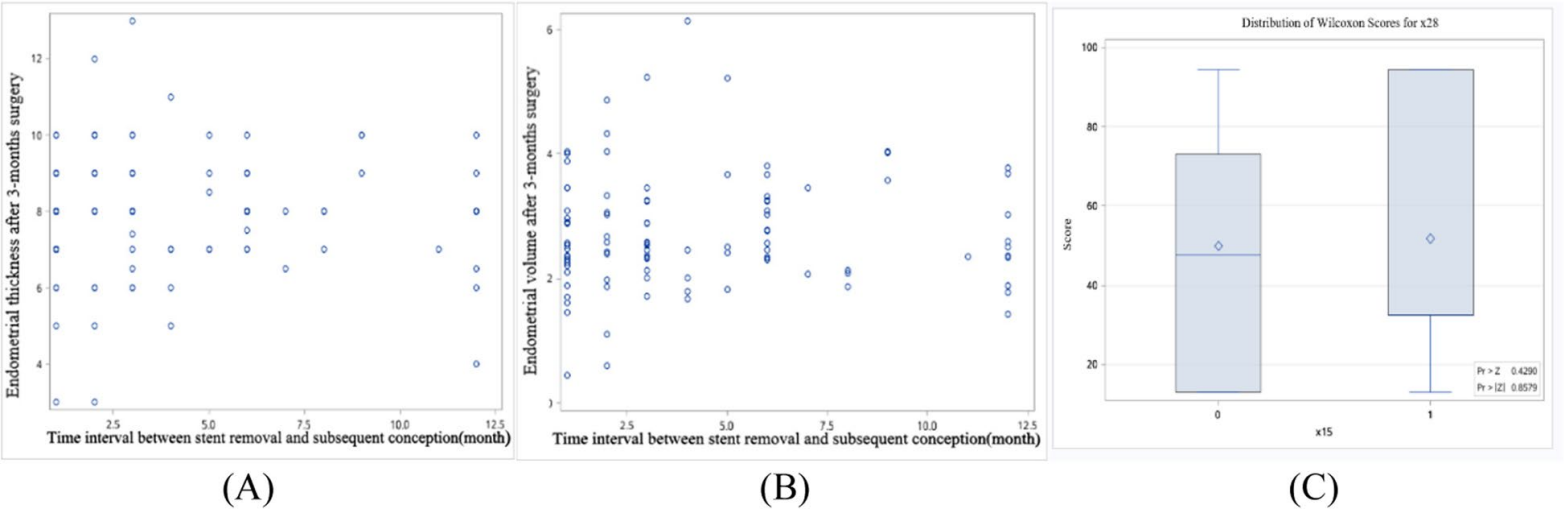
Analysis the factors after 3-months surgery affecting the time interval between stent removal and subsequent conception: **A** Scatter plots of the endometrial thickness and the time interval：Pearson correlation *P*=0.9199, Spearman correlation *P*=0.6728; **B** Scatter plots of the endometrial volume and the time interval: Pearson correlation *P*=0.7813, Spearman correlation *P*=0.6654; **C** The relationship between adhesion recurrence and the time interval (*P*=0.4290)

**Fig. 7:**
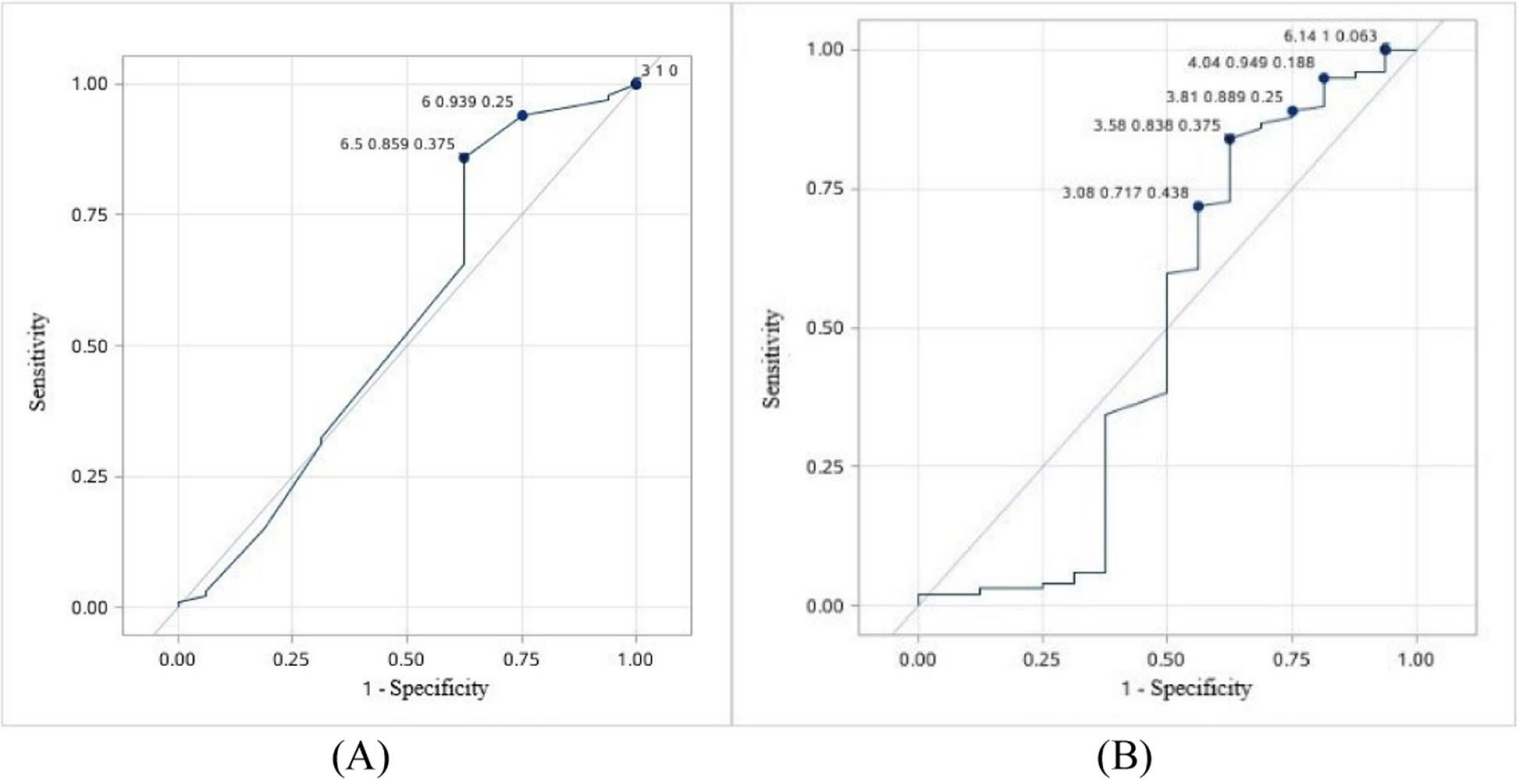
The ROC curve of the endometrial thickness and volume after 3-months surgery predication model for getting pregnant in one year: **A** The ROC curve of endometrial thickness; **B** The ROC curve of endometrial volume

There was no factor affecting the time interval between the stent removal and subsequent conception (*P*>0.05) (Fig. 6). The rate of pregnancy in patients (90%, 90/100) with cavity shape repaired at the second-look hysteroscopy was significantly higher than those (60%, 9/15) without repaired (*P*<0.01) (Table 4). Logistic regression analysis showed that adhesion recurrence was the risk factor for pregnancy (*P*<0.01) (Table 5). The ROC curve showed that the rate of pregnancy in one year was as high as 85.9%, when the endometrial thickness measured after 3-months surgery was more than 6.5mm; the rate of pregnancy in one year can be as high as 83.8%, when the endometrial volume measured after 3-months surgery was more than 3.58cm^3^ (Fig. 7). Regretfully, it had no predictive value. Analysis of the risk factors for miscarriage found that only maternal age was positively associated with miscarriage (*P*<0.05) (Table 6).

**Table 4:** Comparison the general conditions in pregnant patients and non-pregnant patients

	**Pregnant** **(*n*=99)**	**Non-pregnant** **(*n*=16)**	**Rate of pregnancy (%)**	**P**
Adhesion degree before surgery				
Mild	17	4	80.95	0.6867^a^
Moderate	82	12	87.23	
Menstrual blood volume before surgery				
Hypomenorrhea	70	9	88.61	0.2473^a^
Normal	29	7	80.56	
Adhesion recurrence at the second-look hysteroscopy				
No	90	10	90.00	0.0063^a^
Yes	9	6	60.00	
Age	31.00(28.00,34.00)	30.50(28.50,34.00)		0.6153^b^
Gravidity	3.00(2.00,4.00)	2.00(1.50,3.00)		0.2239^b^
Parity	0(0,1.00)	0(0,0.50)		0.2692^b^
Endometrial thickness before surgery (mm)	6.00(5.00,7.50)	6.50(5.00,8.50)		0.4776^b^
Endometrial volume before surgery (cm^3^)	1.89(1.23,2.34)	1.93(1.17,3.34)		0.8178^b^
Endometrial thickness after 3-months surgery (mm)	8.00(7.00,9.00)	8.00(5.50,9.00)		0.5352^b^
Endometrial volume after 3-months surgery (cm^3^)	2.54(2.21,3.25)	2.57(1.333.83)		0.8620^b^

^a^Continuous correction chi-square test

^b^Nonparametric test

**Table 5:** Analysis of the affecting factors for getting pregnant

	**Univariate logistic regression analysis**				**Multivariate logistic regression analysis**			
**Parameter**	***P*-value**	**OR**	**95%CI**		***P*-value**	**OR**	**95%CI**	
Age	0.5747	0.963	0.846	1.097				
Gravidity	0.3397	1.212	0.817	1.799				
Parity	0.3646	1.606	0.577	4.470				
Endometrial thickness before surgery	0.3333	0.889	0.701	1.128				
Endometrial volume before surgery	0.2783	0.779	0.496	1.223				
Endometrial thickness after 3-months surgery	0.3606	1.150	0.852	1.553				
Endometrial volume after 3-months surgery	0.6373	0.894	0.560	1.426				
Adhesion recurrence at the second-look hysteroscopy	0.0041**	0.167	0.049	0.566	0.0041**	0.167	0.049	0.566
Adhesion degree before surgery	0.4552	1.608	0.462	5.591				
Menstrual blood volume before surgery	0.2523	0.533	0.181	1.566				

^*^P<0.05

^**^P<0.01

^***^P<0.001

**Table 6:** Analysis of the affecting factors for miscarriage

	**Univariate logistic regression analysis**				**Multivariate logistic regression analysis**			
**Parameter**	**OR**	**95%CI**		**P**	**OR**	**95%CI**		**P**
Age	1.153	1.025	1.297	0.0181	1.153	1. 025	1.297	0.0181
Gravidity	0.968	0.708	1.324	0.8406				
Parity	1.029	0.473	2.241	0.9421				
Menstrual blood volume after 3-months surgery	1.172	0.447	3.070	0.7468				
Endometrial thickness after 3-months surgery	1.294	0.960	1.744	0.0905				
Endometrial volume after 3-months surgery	1.566	0.961	2.550	0.0716				
Adhesion recurrence at the second-look hysteroscopy	0.593	0.119	2. 942	0.5222				

## Discussion

After intrauterine surgery trauma, infection and other factors lead to the injury of the endometrial basal layer, the surface of the uterine cavity will appear lack of epithelial cell coverage, interstitial exposure, inflammatory cell infiltration and collagen deposition, which will lead to endometrial fibrosis and eventually intrauterine adhesions [19, 20]. Hysteroscopic adhesiolysis is the optimum route for treatment of IUAs [9, 11–13]. To prevent adhesion recurrence after surgery, many approaches have been used in clinic, including amnion graft, cross-linked hyaluronic acid gel, IUD, estrogen therapy and so on [12, 15, 16]. It is reported that the rates of adhesion recurrence were 15.4%-48%, 13.4%-20.2%, 54.3% and 32.6%, respectively, in using amnion graft, cross-linked hyaluronic acid gel, IUD and estrogen therapy after hysteroscopic adhesiolysis [12, 21–24].

Although the mild to moderate IUAs do not lead to amenorrhea, they often lead to repeated early pregnancy loss and even embryo implantation failure due to the characteristic of thin endometrium. Therefore, the patients with fertility requirement also need treatment. As we all know, the degree of adhesion may be aggravated after surgery if there is no effective measure to inhibit scar growth. So, gynecologist is usually cautious to perform surgery for the mild to moderate IUAs, especially for the marginal type of adhesions. In this study, adhesive tissues were separated followed a modified stent placement immediately. It has many potential advantages. Firstly, the endometrial thickness and endometrial volume significantly increased; Secondly, the rate of adhesion recurrence was only 13.04%, lower than that reported in the literatures [12, 21–24]; Thirdly, the pregnancy rate in one year after stent removal was 86.09%, which was higher than those before reported [25–27]. In addition, the operation is easy and convenient without thermal damage to the remaining endometrium [28]. Lastly, the modified stent was available and inexpensive in clinical. Finally, the modified uterine stent is composed of a stainless-steel metal ring and anti-adhesive membrane of Chitosan, which is partially biodegradable in three months and has good mechanical support properties. When placed in the uterine cavity, there is no inflammatory and immunogenic stimulation to the endometrium, and it can be used continuously to prevent uterine cavity contractures. Thus, the scheme of adhesiolysis with a modified stent to treat the mild to moderate IUAs is worth our attention.

In this study, we tried to predict the prognosis of patients by non-invasive ultrasonographic indicators. Which can reflect endometrial receptivity, including endometrial thickness, pattern and blood flow, endometrial echo, peristalsis, volume, and endometrial-myometrial junctional zone [29–32]. It was limited reported in previous studies. We use ultrasound method making the endometrial thickness and endometrial volume numeric vectors, compared with hysteroscopy evaluation, ultrasound measurement endometrial thickness is more objective. It showed that endometrial thickness and volume might reflect the uterine cavity environment, especially the endometrial thickness after 3-months surgery. Because of the combination of antiadhesion membrane and metal ring, the stent can perfectly maintain the uterine cavity shape and inhibit scar contracture within 3 months after surgery. Therefore, it is not necessary to repeat hysteroscopy during this period, only to detect the endometrial thickness by ultrasonography to predict the recovery of the uterine cavity environment. However, when the anti-adhesion membrane is completely dissolved after 3 months, hysteroscopy should be performed in time to remove the bare metal ring that may affect growth of the endometrium or replace a new stent, possible new adhesions were also isolated at the same time. This series of procedures cannot be replaced by ultrasound examination. We suggest that it may attempt to use ultrasonography as a non-invasive method to follow up IUAs after surgery, reduce the number of repeated hysteroscopies. It is believed that with the progress of ultrasound equipment, non-invasive ultrasonographic prediction should be more accurate with more indicators including blood flow, endometrial echo, peristalsis and son on. Non-invasive monitoring after IUA surgery should be standardized.

At present, there is no effective biomedical material to promote endometrial repair and regeneration for the clinical treatment of intrauterine adhesions [19]. The main purpose of the modified stent is to maintain the uterine cavity shape as long as possible to inhibit scar growth until the endometrium itself functional regeneration. The stent does not appear to have the function to directly promote endometrium regeneration. However, we are pleased to discover a significant increase in endometrial thickness after stent placement and a pregnancy rate of 86.09% within one year. Future exploration will be to select suitable biodegradable materials to construct scaffolds or stent loaded with therapeutic drugs or stem cells to enhance endometrial regeneration.

## Data Availability

Data and other materials can be made available by the corresponding author upon a reasonable request.

## Abbreviations

ROC: Receive operating characteristic
IUAs: Intrauterine adhesions
IUD: Intrauterine device
TVUS: Transvaginal ultrasound
AFS: American Fertility Society
